# Implementation of Change Club action plans to promote built environment change in rural communities

**DOI:** 10.1186/s12966-026-01908-x

**Published:** 2026-03-31

**Authors:** Meredith L. Graham, Emma C. Lewis, Deyaun L. Villarreal, Sara C. Folta, Leah C. Volpe, Galen D. Eldridge, Karla L. Hanson, Grace A. Marshall, Jay E. Maddock, Miriam E. Nelson, Rebecca A. Seguin-Fowler

**Affiliations:** 1 Institute for Advancing Health Through Agriculture, Texas A&M AgriLife Research, 17360 Coit Rd, Dallas, TX 75252 USA; 2 Friedman School of Nutrition Science and Policy, Tufts University, Boston, MA 02155 USA; 3 Department of Public and Ecosystem Health, Cornell University, Ithaca, NY 14853 USA; 4 School of Public Health, Texas A&M University, College Station, TX 77843 USA; 5 Friedman School of Nutrition Science and Policy, Tufts University, Boston, MA 02111 USA; 6 Institute for Advancing Health Through Agriculture, Texas A&M AgriLife Research, Norman E. Borlaug Building, 498 Olsen Blvd, College Station, College Station, TX 77843 USA; 7 Department of Nutrition, College of Agriculture and Life Sciences, Texas A&M University, 498 Olsen Blvd, College Station, TX 77843 USA

**Keywords:** Civic engagement, Built environment, Rural, RCT, Change club, Implementation evaluation, CFIR, PSE, Health promotion

## Abstract

**Background:**

Rural United States communities often experience disproportionate burdens of obesity, cardiovascular disease, diabetes, and premature mortality. Built environment constraints, including limited sidewalks, recreation facilities, and access to nutritious foods, may restrict opportunities for adoption and maintenance of healthy eating and physical activity behaviors. Civic engagement approaches empower residents to assess community needs, develop action plans, and implement policy, systems, and environmental (PSE) strategies. However, few randomized trials have examined implementation of PSE strategies and their impacts in rural adult populations. This study evaluated implementation of Cooperative Extension-led Change Club (CC) community project action plans aimed at facilitating PSE change in six rural and micropolitan Texas and New York intervention communities. Presently, we document implementation outcomes and identify factors that may have influenced implementation using the Consolidated Framework for Implementation Research (CFIR).

**Methods:**

CCs followed a 24-module curriculum facilitated by trained Extension educators. Approved action plans were provided with seed money to target diet and physical activity PSE changes. Implementation outcomes regarding the action plans were tracked through educator reports, interviews, and proposals. CFIR factors were assessed during the early stages of action plan implementation using interviews with educators and participant residents.

**Results:**

All six intervention communities implemented action plans, most of which had multiple components. Each prioritized environmental changes and most focused on addressing physical activity. Implementation timing and continuity varied, influenced by external factors such as weather and local approvals. CFIR analysis identified beliefs about feasibility, stakeholder engagement, and group decision-making as key factors influencing implementation.

**Conclusions:**

Rural CCs successfully launched a variety of built environment initiatives. This implementation evaluation highlights pathways and barriers related to scaling rural civic engagement strategies.

**Trial registration:**

Clinical Trial #NCT05002660 (August 2021).

## Background

Rural communities across the United States face persistent health disparities, including elevated rates of obesity, cardiovascular disease, diabetes, and premature mortality, compared with urban and suburban populations [1–3]. These disparities are shaped in part by features of the built environment. Many rural areas lack sidewalks, recreation centers, and safe walking or biking routes [4, 5]. Rural food environments are often constrained by long travel distances to grocery stores and limited access to affordable, nutritious foods [5]. These factors restrict opportunities for physical activity (PA) and healthy eating, which contribute to increased rates of preventable chronic disease [6–8]. Addressing these inequities requires approaches that draw on the insights and leadership of rural residents, who possess critical knowledge of their community’s assets, challenges, and priorities. Empowering residents to lead locally driven efforts can strengthen collective capacity, foster sustainable change, and ultimately promote healthier, more resilient rural communities. One promising strategy for supporting such community-led action is civic engagement.

Civic engagement is defined here as “individual and collective actions to identify and address issues of public concern” [9]. In practice, a group of residents is guided through a structured process of assessing their community, identifying priority issues, and developing and implementing an action plan, which may have multiple components, for policy, systems, or environmental change (PSE) [10]. PSE changes are defined as taking place in one of the following ways: laws or policies (policy), organizational protocols (systems), or physical, social, or economic environments [11]. These approaches have produced measurable improvements in community environments—such as playground and community garden renovations, shade tree planting to encourage walking, creation of walking paths, and installation of pedestrian flashing light signals—and, in some cases, have been associated with improvements in health outcomes among both PSE participants and broader community residents (e.g., increased PA, increased cardiovascular fitness, reductions in blood pressure) [12–20].

An illustrative example is the Healthy Eating and Active Living (HEAL) initiative in Cleveland, Ohio [15]. Through a participatory assessment process, residents identified limited recreation opportunities despite the presence of underutilized neighborhood parks and green spaces. By leveraging community connections, residents recruited volunteer instructors and, within two years, had established more than 40 weekly volunteer-led activities in public parks that reached an average of 300 community members per month. This case underscores how resident-driven, context-specific approaches can mobilize existing community assets to foster sustainable health-promoting changes in the built environment.

Despite the potential of PSE change strategies, rigorous evaluation remains limited. Most PSE projects are not conducted as randomized trials due to ethical, practical, and methodological challenges. In a review of 52 PSE projects focused on improving healthy eating and PA, only two incorporated a randomization component [21]. When randomized PSE trials are conducted, they are often school-based and focused on children or youth [22, 23]. As a result, robust evidence on the impact of PSE initiatives in adult and community settings is lacking, in part due to the complexity of interventions, limited resources for implementation, and insufficient evaluation strategies [21].

A Change Club (CC) is an example of a PSE strategy. A CC is a group of residents (Change Club members [CCMs]) who engage in stepwise planning and implementation of action plans for built environment changes in their communities. The CC curriculum is facilitated by local trained Cooperative Extension educators, and in addition to the stepwise planning, includes group development, needs assessment, and nutrition and PA education. The CC intervention is designed to promote healthy eating and PA behaviors in CCMs and also among other community residents whose behavior may be influenced by CCMs and/or the changes to the local built environment. The CC curriculum has been implemented in several previous studies [13, 14, 24–28]. The current study relies on data from the first randomized trial of the CC [10]. Additional details on the CC curriculum can be found in the protocol paper for the CC randomized trial [10].

Establishing effectiveness alone is insufficient for advancing civic engagement and PSE interventions in rural settings. Given the complexity of community-driven strategies, variability across local contexts, and practical constraints faced by rural communities and Extension systems, it is equally important to understand how these interventions are delivered in real-world settings and what factors influence their success. Implementation evaluation is therefore a key component of rigorous intervention research, helping to explain how and why interventions succeed or fail by examining implementation facilitators and barriers [29, 30]. By complementing effectiveness analyses, implementation evaluations generate insights needed to support adaptation, sustainability, and broader translation across diverse contexts, and are therefore critical for advancing the evidence base for civic engagement and PSE interventions.

Accordingly, the present study focused on examining the implementation of CC civic engagement community project action plans—the PSE interventions delivered within the randomized trial—in rural and micropolitan Texas and New York communities. Specifically, we conducted a content and qualitative analysis to understand which aspects of the CC action plans worked well and why, which factors were challenging and why, and develop recommendations for future implementation. To guide this evaluation, we posed the following research questions:

1. What components of the CC action plans were implemented and when did implementation occur?
2. How were the action plans implemented and what contextual factors, barriers, or facilitators influenced the process?

## Methods

The CC curriculum was implemented using a cluster randomized, two-arm parallel design trial in rural communities (*n* = 12) across New York and Texas. The communities were selected based on lead study investigators’ connections to the Extension systems in New York and Texas, where they are based, which was essential for study execution. Prior to the start of the project, Extension educators (herein, ‘educators’) in qualifying communities were contacted, discussed the project with the lead investigators, reviewed the scope of work for the project, and, if interested, signed on to participate. Communities were classified as rural or micropolitan according to the Rural-Urban Commuting Area version 2.0 definition [31] and designated as medically underserved and/or health professional shortage areas [32]. Recruitment started in May 2022 and ended in April 2023. The study was promoted online (e.g., internet advertisements, advertisements on social media, social media posts), via mailings (postcards and letters), email, encouraging referrals of friends and family members, educator activities, flyers/posters, and traditional media (radio, TV, and newspaper). Randomization occurred at the community level after baseline data collection in both communities of a matched pair, with six communities initiating the CC intervention process immediately and six communities serving as controls. The control communities will be provided with the CC curriculum materials 36 months after baseline (in 2026). This analysis focuses solely on the communities assigned to intervention status (*n* = 6).

### Participants and recruitment

The study enrolled a total of 196 adults to participate as CCMs, 90 of whom were in communities assigned to intervention status. On average, 16 residents per community were recruited to participate in each CC. CCM eligibility criteria required participants to be at least 18 years old and English-speaking, and score ‘poor’ or ‘intermediate’ on at least one item from the American Heart Association’s Simple 7 composite score (e.g., BMI > 25) [33]. More details on eligibility and exclusion criteria are available in the protocol paper [10]. All research activities involving human subjects were reviewed and approved by the Texas A&M University Human Subjects Protection Program (protocol # IRB2021-1490). Participants provided electronic informed consent prior to enrollment.

### Intervention delivery

Educators were trained by members of the study team to facilitate CCs and their regular meetings in their communities. The CC curriculum included 24 modules, delivered in-person, virtually, or in hybrid format, with flexibility to combine modules if needed. There was variability across the communities and within the communities for the frequency and duration of meetings given the flexible structure. Each educator worked with their CCMs to determine their community’s meeting schedule. The curriculum includes an agenda for each module, talking points for the educator, and any relevant handouts, presentations, videos, or other materials. The early sessions in the curriculum emphasized team building and group cohesion, followed by modules on community assessment, advocacy skill development, and asset mapping. CCs then progressed to action planning. The action planning consisted of each CC reviewing a menu of evidence-based PSE changes [34–36] and selecting one or more projects they believed could be feasibly implemented in their community. Examples provided within the curriculum included financial incentives to make healthy foods and beverages more affordable (policy change), structured worksite programs that encourage activity and provide a set time for PA during work hours (system change), and initiating a school garden program (environment change). After completing Module 12, each CC developed an action plan for their community project which could contain multiple components (projects). These plans and their implementation are the focus of this paper. The full curriculum and training materials can be accessed online (https://strongpeopleprogram.org/).

### Implementation tracking and analysis

The study team maintained detailed records to document intervention implementation. Educators submitted action plan proposals using a standardized template that outlined the objectives, action items, stakeholders, budget justification, and anticipated timeline for each community’s selected PSE project components. The study team reviewed submitted proposals for feasibility, sustainability, and likelihood of execution, requesting revisions when necessary. Once approved, communities received up to $5,000 in seed money from the study team to support implementation. The actual total amounts spent by each CC ranged from $2,200 to $5,000. Progress on action items was subsequently discussed during regular calls between educators and the study team, and activities were logged to compile a record of action plan implementation and timing.

At 12 months since initiating the curriculum, three study team members independently reviewed the action plan implementation timing and study notes, and coded implementation progress using three categories: ‘Significant action plan component(s) completed’; ‘Partial action plan progress but delayed’; and ‘Minimal or no action plan progress’. The three study team members discussed scores and any discrepancies were rectified. The team met again to revisit the assessment of CC progress at 24 months past initiation of the curriculum and there were no discrepancies in the scoring for each CC. The team also applied criteria for the inclusion of CC activities in the assessment of progress. The activity must have been specified as a component within the CC’s action plan, and it must have exceeded the standard work plan for the CC educator (for example, a walking group promotion that the educator conducted annually in the CC community prior to the formation of the CC was not considered in the assessment of CC progress).

In addition, action plans were reviewed by one study team member and coded using the following categories: (1) PSE level, where ‘policy’ indicated whether the change advocates for and promotes the passage and implementation of policies that are designed to impact diet and/or PA behaviors, ‘systems’ indicated whether the change involves changing the rules or infrastructure within an organization to encourage healthier choices, and ‘environmental changes’ indicated whether the change includes structural improvements or new programs and services; (2) Domain, which was categorized as either ‘nutrition’ or ‘physical activity’; (3) Setting, which was categorized as ‘public library’ or ‘senior center’ or ‘businesses’ if at least one project component of the action plan took place in those locations, and ‘community’ if it took place in public community spaces such as parks; (4) Evidence-based examples which were examples from evidence-based repositories that aligned with the project component; and (5) CC curriculum examples which were project components from previous CC action plans that were included in the curriculum and aligned with the project component. A second study team member reviewed the coding and any discrepancies were rectified.

### Implementation interviews

A study team member interviewed each educator (*n* = 6) and 1–2 CCMs from each community (*n* = 9) after each community completed the curriculum. CCMs were eligible to participate in the interviews if they consented to participate and be recorded, did not opt out of the study or meetings, attended greater than 30% of meetings, and attended at least one meeting in the two months prior to completing Module 24. CCMs were selected to participate using a randomly generated rank order. Educators and CCMs were invited to participate in the interviews via email. Interviews were scheduled for 60 min; CCMs received a $40 gift card for their participation. Educators did not receive a gift card for their participation. Semi-structured interview guides, informed by the Consolidated Framework for Implementation Research (CFIR) [30], were developed for both educator and CCM interviews. The CFIR framework was pre-specified within the grant proposal as the framework for the process evaluation since it offered a structured approach that was well suited for the multilevel and dynamic nature of CC changes over time. Each domain was assessed for relevance to the study, potential to act as a barrier or facilitator, and sufficient likelihood for variation across sites [30]. This assessment was conducted by one study team member and brought to the overall team for input and discussion. All interviews were conducted virtually using Zoom teleconferencing software, which automatically generated verbatim transcripts. Transcripts were manually reviewed for accuracy against the audio recording and then de-identified. Qualitative interview data were analyzed using a directed content analysis approach, drafting an initial codebook based on the interview guide [37]. A codebook was developed to align with CFIR domains in the interview guides [29, 30]. The CFIR domains are defined as: (1) Adaptability, which refers to thoughts about how rigid or adaptable the action plan is, especially to address unforeseen barriers); (2) Beliefs about CC action plan, which relates to the potential impact of the action plan on the community and whether it is worthwhile to do; (3) Engaging, which are the reasons that CCMs gave for participating; (4) Evidence strength and quality, which is the information gathered to choose the action plan project component(s); (5) Implementation climate, which is also known as “tension for change”—while CCs see a need for the project, what is the perception about or evidence that the community sees a need for it?; (6) Intervention source, which is the process by which the group came to a decision—who in the group brought forward the idea for the chosen project or who championed the idea?; (7) Readiness for implementation, which are the perceptions about the resources needed and cost of the project, how those will impact feasibility of the action plan, and how those impacted the decision to choose the project; (8) Relative advantage, which is how the CC action plan compares to other initiatives in the community; and (9) Collective efficacy, which is confidence in the group being able to complete the action plan [29, 30].

NVivo (QSR International, Doncaster Australia; Version 13, Release 1.7.2) was used to assist with coding. To assess consistency and determine inter-coder reliability, as an initial step, three members of the study team each independently coded a randomly selected transcript. This revealed minor discrepancies which were discussed. The codebook was then refined, mainly by clarifying code definitions. Subsequently, each of the three coders independently coded 10 transcripts such that all transcripts were double-coded. Cohen’s kappa was 0.7 or greater at all codes, indicating substantial agreement [38]. We then examined data for common themes based on frequencies of similar responses and patterns found in the data. Quotes were identified that represented the essence of each theme or that illustrated the variation in perspectives.

## Results

Action plans were implemented across all six communities (Table 1). We considered each community to have one overall action plan, and most action plans had more than one component. All action plan components were classified as environmental changes. Most components of the action plans aimed to address PA (*n* = 11), while fewer addressed diet (*n* = 2) or combined both domains (*n* = 2). PA-focused action plans were implemented across varied community settings, including libraries (*n* = 2), a senior center (*n* = 1), businesses (*n* = 1), and broader community venues (*n* = 11).

**Table 1:** CC action plan project components classified by behavioral domain and setting

CC Action Plan Project Components Implemented	Domain	Setting	Evidence-Based Example	CC Curriculum Example
Local Businesses’ Walking Groups (*Walk Across Texas!*)	PA	Businesses	Community-based social support for PA [39]	Previous walking group CC project
Physical Activity Library Equipment Rental (e.g., snowshoes)		Public Library	Places for PA [40]	Improve accessibility of recreation and exercise spaces and facilities (e.g., building parks or playgrounds, increasing operating hours, using school facilities during non-school hours)
Library PA Equipment Rental (e.g., balls, bats)				
Open Gym Nights		Community		
Park Trail Signs				
Trail Markers, Benches, Dog Waste Receptacles				
Park Map				
Pickleball League			Recreational sports leagues for adults [41]	
Offering StrongPeople ^®^ Strong Bodies Fitness Program			Activity programs for older adults [42]	
Annual 5 K Mud Run			Community-wide PA campaigns [43]	Previous 5 K CC project
Walking Passport				Previous walking brochure CC project
Booth at Community Health Fair	Both (PA & Diet)			Previous health fair CC project
Online Healthy Events Community Calendar			Mass media campaign for PA [44]	Previous health directory CC project
Community Fridge	Diet		Food hubs [45]	Previous roadside food pantry CC project
Senior Center Raised Garden Beds		Senior Center	Community garden [46]	Previous community garden CC project

**Fig. 1:**
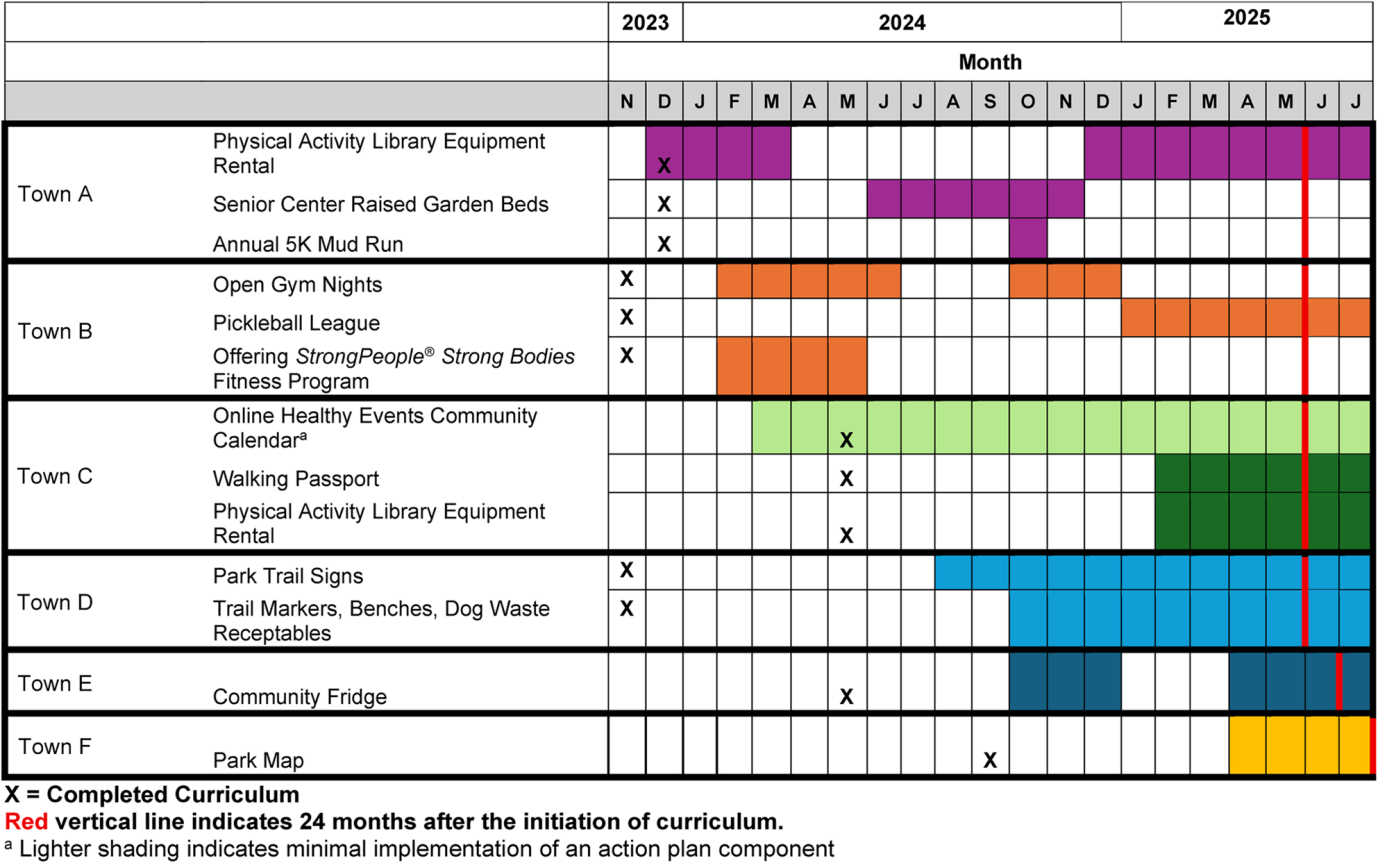
CC action plan implementation and timing

In each community, at least one component of their action plan was implemented within one year of completing the curriculum (Fig. 1). However, the timeline of action plan implementation varied substantially across the six intervention communities. CCs in two towns, Town A and Town B, completed the overall curriculum and commenced implementation of their action plans early in 2024. These two CCs also included multiple components as part of their action plans, including fitness programs, equipment rentals, and community events, with several initiatives sustained across 2024–2025. In contrast, Town C and Town D implemented project components more gradually, rolling out informational strategies (e.g., online calendar, trail signs) earlier and built environment improvements (e.g., equipment rental, benches) later in 2024, with activities sustained into 2025. Town E and Town F were the last communities to implement their action plans, each initiating 1–3 project components in late 2024 or 2025. Across communities, some project components were environmental assets (e.g., raised garden beds, trail markers, community fridge), while others were discrete or seasonal events (e.g., annual Mud Run). Collectively, Fig. 1 depicts how action plans unfolded differently over time, with some communities able to implement multiple sustained initiatives while others launched fewer or more time-limited activities.

At 12 months after initiation of the curriculum, the study team rated Town A and Town B as having ‘significant action plan component(s) completed’; three (Town C, Town D, and Town E) as having made ‘partial action plan progress but delayed’; and Town F as ‘minimal or no action plan progress’. At 24 months after initiation of the curriculum study team members were in consensus that the categorizations remained accurate except that Town F had completed an action plan component and could now be considered as ‘partial action plan progress but delayed’.

Qualitative analysis of CFIR constructs showed both commonalities and differences across communities (Table 2). Adaptability of action plans was noted across communities and did not vary much by level of implementation progress. Beliefs about the action plan differed: communities with significant progress (Town A, Town B) reported more concerns about feasibility and workload, whereas partial and minimal progress communities generally expressed enthusiasm without major concerns. Across communities, reasons for engagement most often emphasized health and fitness, with “helping the community” more frequently mentioned in partial progress communities. Research study incentives were rarely described as a primary motivator. Stakeholder engagement was reported by all communities when selecting project components, while community audits were mentioned less often but evidence strength and quality did not clearly impact implementation. The implementation climate varied across communities but was not strongly associated with implementation progress. Variation was evident in intervention source processes. Communities with significant action plan components completed described longer deliberation and consensus-building, while other communities reported stalled decision-making or conflict. Readiness for implementation was partly shaped by the $5,000 seed money, which guided project component selection and scope. There were mixed perceptions related to relative advantage with no apparent relationship with implementation. Collective efficacy was reported as high across communities, though timeline concerns were more common in delayed progress communities.

**Table 2:** CFIR constructs influencing CC action plan implementation

CFIR Construct	Summary of Findings Across Communities	Supporting Quotes	Relation to Implementation
Adaptability	Action plans were generally viewed as flexible; some minor adjustments were made. Not a key barrier.	*“I’d say we’re keeping to [the plan] very well*,* I think we have a doable timeframe and action plan*,* but*,* yeah*,* like the wiggle room with the actual times…” (CCM)*	Similar across all communities; not a differentiator.
Beliefs about the Action Plan	Mixed views. More successful communities expressed more concerns (workload, feasibility). Less successful communities were more uniformly positive.	*“…I will definitely be surprised if we get a significant number of people that show up.” (CCM*,* substantial success community)* *“I think it’s a good plan. And it’s gonna provide opportunities for people.” (CCM*,* partial success community)*	Enthusiasm did not guarantee implementation success.
Engagement (i.e., Reasons for participation)	Across communities, participants were motivated by health and fitness. “Helping the community” was more often cited at less successful communities. Incentives provided for data collection were not a primary motivator.	*“Just general improved health*,* exercise*,* better lifestyle [caused me to be interested].” (CCM)*	Intrinsic motivation drove ongoing engagement more than incentives.
Evidence Strength and Quality	All communities engaged stakeholders (e.g., councils, organizations) in selecting project components. Community audits rarely influenced decisions.	*“We went to the town council meetings*,* going to the recreation department*,* going to the [local conservancy]*,* really trying to figure out*,* well*,* what does [town] need?” (Educator)*	Stakeholder engagement common; did not clearly differentiate implementation success.
Implementation Climate	Perceptions of community need varied. Some communities were confident of demand (e.g., food pantry use, active trail users), others uncertain.	*“Nevertheless*,* as to whether or not they’re going to make use of [our project]*,* we just don’t know.” (Educator)*	Varied by community but not strongly tied to implementation success.
Intervention Source	All groups followed curriculum; differences emerged in how ideas were proposed, debated, and resolved. More successful communities took longer but achieved consensus.	*“We all kind of kept going round and round and round. And we just kept talking to more people and talking. And that’s where it was getting frustrating for a lot of us*,* because we would come and meet for 2 hours*,* and we would be talking about the same thing over and over and over and over and over again… So*,* that was hard.” (CCM*,* substantial success community)* *“We came up with three different ideas and then settled on [one]… And the final decision was left with that one.” (CCM*,* minimal success community)*	Extended deliberation may have facilitated stronger implementation.
Readiness for Implementation	The $5,000 seed money strongly influenced project component choice; groups limited ideas based on budget. Some described dismissing “grand” ideas due to cost/time.	*“Well*,* we knew that the scope of the project had to be limited in terms of -- it couldn’t be too large*,* because we knew what the parameters were in terms of the budget.” (CCM)*	Communities that matched project components to financial resources moved forward more smoothly.
Relative Advantage	Mixed perceptions. Some viewed CC project components as unique; others as complementary to existing efforts.	No representative quotes, given mixed perceptions.	No clear pattern across implementation success.
Collective Efficacy	Confidence was generally high, though timeline concerns were noted at less successful communities. Belief in members’ skills and partnerships contributed to confidence.	*“But I think as a group*,* we do have some very good*,* good people in the group that can get stuff done.” (CCM)*	Confidence was present across communities; not a clear differentiator.

Approval or support from local authorities and weather conditions emerged as important contextual factors that contributed to decisions to move forward with specific action plan components and delays in other action plan components. For example, one CC originally wanted to create a dog park, but could not garner support from village leadership, so could not determine a location for the park; ultimately, the group chose a completely different direction for their action plan. Another CC wanted to revitalize their senior center but ran into political issues when trying to work with the county on that plan; this CC also ultimately decided on a completely different action plan. Another CC wanted to build raised garden beds in community playgrounds, but city officials were not on board with the idea, as they worried about vandalism and responsibility for ongoing maintenance; this CC’s action plan evolved into something the city was already interested in supporting. Weather also delayed action plan components. Initiation of a snowshoe rental program at a public library was delayed because, although snowshoes had been purchased and there was a display about the program at the library, there was an atypically low level of snowfall in the area. For another CC, installation of signs and markers was delayed due to frozen ground, making it impossible to dig holes for trail signs until late spring.

## Discussion

This evaluation offers initial insights into the implementation of civic engagement PSE interventions in rural and micropolitan communities, launched in the context of a randomized trial. Across six intervention communities in Texas and New York, CCs developed and initiated action plans that addressed PA and nutrition environments. However, implementation varied in timing, continuity, and scope, with some project components established as sustained environmental assets (e.g., raised garden beds, community fridge, trail signage) and others delivered as discrete or seasonal events (e.g., annual Mud Run, health fair booths). These findings highlight how delivery timelines, group dynamics, and contextual barriers shape resident-led action for PSE change in rural settings.

Communities successfully launched diverse built environment projects but faced interruptions in timing and delivery. Continuity was also an issue in a recent multicomponent sports-based afterschool intervention [47]. With the CC, starts and stops were common, stemming from contextual factors such as weather (e.g., delayed installation of signage in freezing conditions) and challenges obtaining approval from local authorities for park and trail improvements. These challenges underscore the reality that these initiatives operate within broader structural constraints that require flexibility and persistence.

From a CFIR perspective, several constructs provided insight into influences on implementation. While adaptability was not a differentiating factor, beliefs about the action plan, engagement motivations, and intervention source processes appeared to shape implementation trajectories. Interestingly, the two communities that completed implementation earlier were more likely to deliberate extensively and express concerns about feasibility, whereas communities with delayed progress tended to describe enthusiasm without substantial critique. This pattern suggests that constructive skepticism and critical dialogue may facilitate more realistic planning and greater follow-through. In an evaluation of SWITCH, a school-based wellness intervention, researchers also found that reflecting and evaluating as part of the intervention process were associated with greater intervention fidelity and adoption [48]. Across communities, stakeholder engagement was central to project selection, yet community audits were rarely used as a guiding tool. This finding aligns with prior research showing that residents often value lived experience and existing relationships over formal assessments when making decisions about community priorities [49–51].

Several lessons and recommendations emerge for future implementation of PSE strategies in rural contexts. First, implementation timelines may need to be adjusted to account for delays related to weather or approval processes allowing groups additional flexibility, and techniques should be developed to help them maintain momentum during lulls in activity. This may include virtual planning in online discussion groups or shared leadership between the educator and CCM(s). Second, stakeholder partnerships should be complemented with practical tools for capacity-building, such as fundraising guidance, to help communities pursue projects beyond seed money limits. Third, structured tools like community audits may need to be redesigned or repositioned to feel less like “research” and more like a practical planning aid to ensure meaningful use by participants. Fourth, all of the interventions chosen were environmental interventions. This may have been due to the appeal of creating tangible products or related to perceived barriers to policy and systems changes in their rural communities. However, policies and systems changes could create more lasting impacts [52].

Importantly, there are also several limitations to consider when interpreting these findings. This implementation evaluation is limited to two data sources (study records and qualitative interviews), neither of which are observed data. This evaluation was also unable to estimate the extent to which the project components were used by residents or the perceptions of residents related to these project components. Lastly, while this analysis captured project implementation at an interim follow-up, longer-term outcomes related to behavior change or health improvements have not yet been assessed.

## Conclusions

This implementation evaluation demonstrates that rural CCs were able to identify priorities, mobilize partnerships, and implement diverse built environment projects within their communities. The evaluation underscores both the promise and the challenges of PSE strategies: while action plans were carried out in all intervention communities, their delivery was heterogeneous and shaped by contextual barriers such as weather and the need for town approvals. Together, these findings highlight the value of implementation evaluations for understanding not just whether community-driven interventions are implemented, but how timing, stakeholder engagement, and contextual conditions influence their progress. Future PSE efforts should incorporate enhanced capacity-building support, flexibility in delivery timelines, and proactive communication strategies to improve implementation, visibility, and sustainability. Longer-term follow-up will be essential to assess the enduring impacts of civic engagement approaches on community health outcomes in rural settings.

## Data Availability

The datasets used and/or analyzed during the current study are available from the corresponding author on reasonable request.

## Abbreviations

BMI: Body Mass Index
CC: Change Club
CCM: Change Club Member
CFIR: Consolidated Framework for Implementation Research
HEAL: Healthy Eating and Active Living
PA: Physical Activity
PSE: Policy, Systems, and Environmental
